# Observation and Characterization of the Hg‐O Diatomic Molecule: A Matrix‐Isolation and Quantum‐Chemical Investigation

**DOI:** 10.1002/chem.202202740

**Published:** 2022-12-14

**Authors:** Lester S. Andrews, Yetsedaw A. Tsegaw, Han‐Gook Cho, Sebastian Riedel

**Affiliations:** ^1^ Department of Chemistry University of Virginia Charlottesville Virginia 22904 USA; ^2^ Anorganische Chemie Institut für Chemie und Biochemie Freie Universität Berlin Fabeckstrasse 34–36 14195 Berlin Germany; ^3^ Department of Chemistry Incheon National University 119 Academy-ro, Yeonsu-gu Incheon 22012 South Korea

**Keywords:** Hg−O diatomic molecule, matrix-isolation spectroscopy, mercury, ozonides, superoxide

## Abstract

Mercuric oxide is a well‐known and stable solid, but the diatomic molecule Hg−O is very fragile and does not survive detection in the gas phase. However, laser ablation of Hg atoms from a dental amalgam alloy target into argon or neon containing about 0.3 % of ^16^O_2_ or of ^18^O_2_ during their condensation into a cryogenic matrix at 4 K allows the formation of O atoms which react on annealing to make ozone and new IR absorptions in solid argon at 521.2 cm^−1^ for Hg‐^16^O or at 496.4 cm^−1^ for Hg‐^18^O with the oxygen isotopic frequency ratio 521.2/496.4=1.0499. Solid neon gives a 529.0 cm^−1^ absorption with a small 7.8 cm^−1^ blue shift. CCSD(T) calculations found 594 cm^−1^ for Hg^16^O and 562 cm^−1^ for Hg^18^O (frequency ratio=1.0569). Such calculations usually produce harmonic frequencies that are slightly higher than the anharmonic (observed) values, which supports their relationship. These observed frequencies have the isotopic shift predicted for Hg−O and are within the range of recent high‐level frequency calculations for the Hg−O molecule. Spectra for the related mercury superoxide and ozonide species are also considered for the first time.

## Graphical abstract

**Mercury atoms** from laser ablation of a dental amalgam target react with oxygen atoms upon co‐condensation in excess argon and neon, and they provide frequencies of 521.2 and 529.0 cm^−1^, respectively, which support high‐level calculations for this weakly bound Hg−O diatomic molecule. The small difference between our neon and argon matrix observations for Hg−O suggest a slightly ionic molecule. Spectra are also observed for Hg‐O_2_ and Hg‐O_3_.

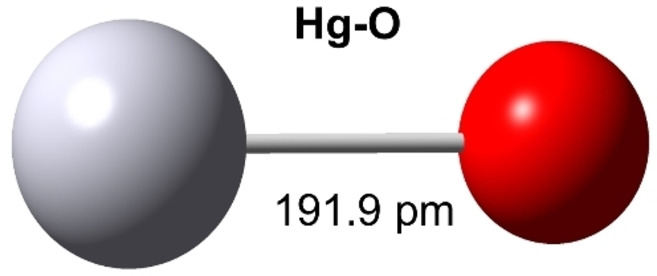

## Introduction

The diverse chemical properties of mercury have attracted considerable attention in the scientific community, ranging from industrial applications to toxicology.^[1]^ Solid mercuric oxides with the −Hg−O−Hg−O‐zigzag chain structures have a broad 500 cm^−1^ infrared absorption band.[^2^, ^3^] Recent high‐level calculations documented a harmonic vibrational frequency between 500 to 600 cm^−1^ for the molecular ^1^Σ^+^ Hg−O, depending on the methods employed.[^4^, ^5^, ^6^, ^7^] Further theoretical studies on this molecule are also available to address the depletion of gaseous mercury in the Earth's atmosphere.[^7^, ^8^] However, owing to calculations at the CCSD(T) level of theory, which revealed a low dissociation energy of 4 kcal/mol to the ground‐state atoms[^4^, ^7^] other physical parameters for this weakly bound Hg−O molecule were difficult to obtain.^[9]^ Therefore, the Hg−O molecule containing common elements is a rare unobserved diatomic molecule.[^10^, ^11^] New argon matrix frequencies at 521.2 cm^−1^ for Hg−^16^O and at 496.4 cm^−1^ for Hg−^18^O correlate very well with these theoretical and solid frequencies and with the mass dependence of the 1.0569 isotopic oxygen 16/18 frequency ratio. Solid neon gives a 529.0 cm^−1^ frequency for Hg−^16^O and at 503.2 cm^−1^ for Hg−^18^O (frequency ratio=1.0513). A pure oxygen matrix exhibits similar features at 522.7 cm^−1^ for Hg−^16^O. Mercury atoms were laser ablated from a dental amalgam target for these reactions with O_2_ and its atomic dissociation products.^[13]^ The Hg resonance radiation from the ablation process supports dissociation of O_2_ into O atoms which react with Hg to form Hg−O on sample annealing. Infrared spectra will also be presented here for the related molecular HgO_2_ and HgO_3_ species.

Previously, laser ablated Zn atoms were reacted with O_2_ to make isotopic zinc monoxide (Zn−O) molecules trapped in argon (769.2 cm^−1^ for ^64^Zn−O).^[12]^ Likewise, cadmium monoxide (Cd−O) was detected at 645.1 cm^−1^ in the argon matrix.^[12]^ This approach using a dental amalgam target for laser ablation of mercury^[13]^ will be employed here to form the diatomic Hg−O molecule. Note that elemental Hg cannot be laser ablated, as Hg atoms would only be removed at much lower temperature. Pulse laser ablation at a solid amalgam target produced excited Hg atoms for our experiments. This amalgam solid was prepared by mixing Hg with the dust of several heavy metals, all donated by a local dentist as described in detail in a preceding work.^[13]^ Recent high‐level calculations in particular by Shepler and Peterson in 2003 and 2007,[^4^, ^5^] provided evidence of the harmonic frequency for the ^1^Σ^+^ ground state of Hg−O. The computed frequency at the CCSD(T) level of theory in all‐electron calculations ranges 512–550 cm^−1^ (nonrelativistic) or 566–601 cm^−1^ (Douglas‐Kroll) depending on the treatment of relativistic effects.

## Results and Discussion

### Hg‐O

We performed CCSD(T) calculations using the Dunning basis set, which found 594 cm^−1^ for Hg^16^O and 562 cm^−1^ for Hg^18^O (ratio 1.0569). Such calculations usually produce harmonic frequencies that are slightly higher than the observed anharmonic frequencies. These calculations demonstrate that our 521.2 cm^−1^ absorption band is in the right place for Hg−O, and its observed 16/18 isotopic frequency ratio 521.2/496.4=1.0499 is also in the expected relationship with our ratio calculated as 594/562=1.0569 from the harmonic oxygen isotopic mass dependence, which is slightly higher than the observed anharmonic frequency ratio (1.0499) (Table 1).

**Table 1:** Calculated and experimentally observed IR frequencies (cm^−1^) of HgO.

	16O	18O	Ratio (16/18)
CCSD(T)^[a]^	594	562	1.0569
Expt Ne	529.0	503.2	1.0513
Expt Ar	521.2	496.4	1.0499

[a] aug‐cc‐pVTZ‐PP basis sets.

**Figure 1:**
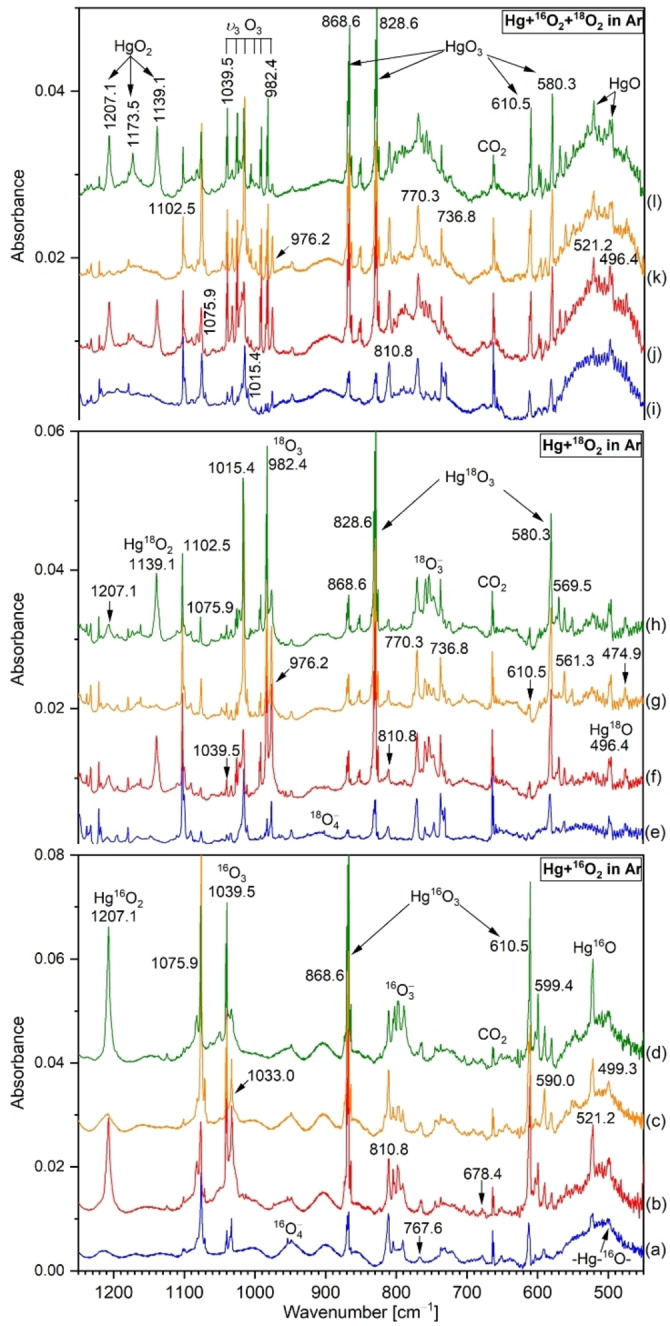
IR spectra of the reaction products from laser ablated Hg co‐deposited with 0.3 % O_2_ (bottom), 0.3 % ^18^O_2_ (middle) and 0.3 % ^16^O_2_+^18^O_2_ (1 : 1) (top) in argon at 4 K. Spectra (a, e, i) after deposition for 60 min at 4 K, (b, f, j) after annealing to 25 K, (c, g, k) after 15 min full‐arc photolysis with mercury lamp and (d, h, l) after annealing to 30 K.

New spectra of the major product from the reaction of Hg atoms produced by laser ablation from mercury amalgam tooth filling material reacted with ^16^O_2_ (0.3 %), with ^18^O_2_ (0.3 %) and with a 1 : 1 mixture of ^16^O_2_+^18^O_2_ (0.3 %) in an argon matrix at 4 K are shown in Figure 1. Results in neon and oxygen matrices are also provided in Figures S1 and S2. The products obtained are compiled in Tables 2 and S1. These new bands at 521.2 and 496.4 cm^−1^, respectively, with the 1.0499 ratio, increased slightly on sample annealing. Similar experiments with CO produced a new band at 1941.6 cm^− 1^ (with C^16^O) and at 1894.7 cm^−1^ (with C^18^O) (frequency ratio=1.0248, the C atom does a lot of the moving there) as well as a weak band at 521.2 cm^−1^ increased ten‐fold on annealing to 25 K. It is important to realize that the 521.2 cm^−1^ band remains sharp while it increases on annealing the solid argon to 25 K, which allows diffusion of oxygen atoms and facilitates their reactions with other atoms in the matrix, particularly Hg. Concurrently absorptions for ozone (v_3_) appear at 1039.5 cm^−1^ on sample deposition and increase markedly on annealing, but decrease on mercury arc photolysis (Figures 1 and S3, Table S2).[^14^, ^15^] Their ^18^O_3_ counterpart at 982.4 cm^−1^, is separated by 57.1 cm^−1^, and defines their isotopic frequency ratio, 1.0581, which is close to that for a pure oxygen motion, 18/16=1.125. Figure 1 shows strong additional counterpart bands at 1075.9 and 1015.4 cm^−1^ (ratio=1.0585) above the very strong ozone ν_3_ mode, that is formed in the O+O_2_ reaction. These new bands at 1075.9 and 1015.4 cm^−1^ behave in the opposite way to the ozone bands; they increase upon photolysis and could not be clearly assigned to any species so far. However, they follow the isotopic pattern of ozonides and could be associated with this species.

**Table 2:** IR frequencies (cm^−1^) of mercuric oxide, superoxide and ozonide species isolated in argon, neon and oxygen matrices at 4 K.^[a]^

Species	Argon			Neon			Oxygen
	^16^O_2_	^18^O_2_	Ratio (16/18)	^16^O_2_	^18^O_2_	Ratio (16/18)	^16^O_2_
HgO	521.2	496.4	1.0499	529.0	503.2	1.0513	522.7
HgO_2_	1207.1	1139.1	1.0597	1220.0	1159.0	1.0562	1209.3
HgO_3_	868.6	828.6	1.0483	872.6	833.9	1.0465	870.8
	610.5	580.3	1.0520	619.8	589.0	1.0523	610.2

[a] The complete product assignment is given in Tables S1 and S2 of the Supporting Information.

Figure 1 (top, spectra (i) to (l)) also suggests that a single ^16^O atom is present in the new molecule responsible for the sharp 521.2 cm^−1^ argon matrix absorption since this band does not change when a mixture of ^16^O_2_ and ^18^O_2_ is used in the experiment and its growth on annealing would include some structure if a mixed isotopic 16,18 product were produced. Similar features are observed in solid neon (Figure S1). The top neon spectra with ^18^O_2_ follow the bottom neon spectra using ^16^O_2_ which gives approximately equal ^16^O and ^18^O vibrational bands in this experiment. Only sharp single pure isotopic bands are observed at 529.0 and 503.2 cm^−1^ in the neon matrix (isotopic ratio 1.0513). The difference between isotopic frequencies is 24.8 cm^−1^ in solid argon and 25.8 cm^−1^ in solid neon which has sharper, better resolved bands for Hg−O (Figure S1, Tables 2 and S1). Thus, new argon and neon matrix spectra enable their assignment to the diatomic Hg−O molecule. The small difference between our neon and argon matrix observations for Hg−O suggest a slightly ionic molecule.^[11]^ Molecular Hg−O was also detected in a pure oxygen matrix at 522.7 cm^−1^ (Figure S2, Tables 2 and S1). Finally, it is worth mentioning that our spectra in solid argon, neon and oxygen also show a broad IR band around 500 cm^−1^ corresponding to the absorptions of polymeric mercuric oxide.[^2^, ^3^]

### HgO_2_

The situation is different for the 1207.1 and 1139.1 cm^− 1^ bands which also increase significantly on annealing: They develop a weaker intermediate component increasing at 1173.5 cm^−1^. This spectrum is shown in the upper left corner of Figure 1 which requires a contribution from ^18^O_2_ for the two lower bands in this isotopic triplet at 1207.1, 1173.5 and 1139.1 cm^−1^. The first band at 1207.1 cm^−1^ requires ^16^O_2_ (Figure 1, bottom) and 1193.1 cm^−1^ needs ^18^O_2_ (Figure 1, middle) and the central band at 1173.5 cm^−1^ requires both isotopes as it is due to Hg^16^O^18^O with Hg reaction (Figure 1, top). The central band is weaker here since it must form in the matrix (from ^16^O and ^18^O atom dissociation products) and ^16^O_2_ and ^18^O_2_ react straightaway with Hg on deposition. The comparison with our previous work on LiO_2_ makes this obvious because those experiments^[16]^ employed a 20/50/30 % mixture of ^16^O_2_, ^16^O^18^O and ^18^O_2_ and those band intensities followed the abundance of the precursor O_2_ isotopes whereas in the present experiments ^16^O^18^O was lower in abundance because it had to be made on deposition using ^16^O and ^18^O from dissociation of their diatomic molecules.

We now compare LiO_2_ and HgO_2_: Both contain the superoxide O−O stretching mode, which is 1207.1 cm^−1^ for HgO_2_ and 1096.9 cm^−1^ for LiO_2_.^[16]^ The higher frequency for the mercury species is due to the higher ionization energy for Hg and reduced ionicity as compared to Li‐O_2_. The most significant bands for ^7^Li^16^O_2_ are the O−O stretch at 1096.9 cm^−1^, which downshifts 61.7 cm^−1^ for ^7^Li^18^O_2_, and up 0.5 cm^−1^ for ^6^Li^16^O_2_ and the ^6^Li^18^O_2_ stretch shifts down 61.5 cm^−1^ for the ^7^Li counterpart.^[16]^ These modes provide an example for Hg and O_2_: The 1207.1 cm^−1^ band shifts down 68.0 cm^−1^ to 1139.1 cm^−1^, which is assigned to an O−O stretching mode (*v*_1_). Thus, the 1207.1, 1173.5, and 1139.1 cm^−1^ oxygen isotopic triplet is assigned to isosceles triangular Hg−O_2_. The 1207.1/1139.1 ratio 1.0597 represents an almost pure O−O stretching mode. The first isosceles triangular molecule Li‐O_2_ exhibited this O−O mode at 1096.9 cm^−1^ with a 16/18 oxygen frequency ratio of 1.0596.^[16]^ The relative intensities reflect the amount of the mixed isotopic molecule ^16^O^18^O in the sample. Such isotopic isosceles triangular molecules are common to both alkali and alkaline earth metal superoxides, but now reported for the first time for mercury species.[^17^, ^18^, ^19^]

It is noteworthy that the broad 500 band for solid Hg−O is about the same in neon or argon matrices, but the triplet for Hg^16^O_2_, Hg^16^O^18^O, Hg^18^O_2_ in argon is only a broad isotopic doublet for Hg^16^O_2_ and Hg^18^O_2_ in neon. In solid neon we do not observe the Hg^16^O^18^O species. This indicates that the neon matrix is not able to stabilize ^16^O_2_−^18^O_2_ for its reactive photodecomposition to ^16^O^18^O like the more rigid argon matrix can. In argon matrices a broad band at 1210 for Hg^16^O_2_ with a broad shoulder at 1140 for Hg^18^O_2_ is shown. In contrast ozone gives sharp bands at 1039.5 in argon and 1041.5 in neon.

### HgO_3_

Figure 1 shows the strongest new product bands at 868.6 and 610.5 cm^−1^ (spectra (a) to (d)) for the ^16^O_2_ investigation and at 828.6 and 580.3 cm^−1^ (spectra (e) to (h)) for the analogous ^18^O_2_ experiments, which increases significantly upon annealing of the argon matrix to 25 K. Since an excess of ozone and mercury atoms is produced in our experiments, it is tempting to assign these bands to HgO_3_. Previous studies on solid ozonides whose crystal structure is well determined, such as KO_3_ ^[20]^ and our recent work on [NEt_3_Me][O_3_],^[21]^ show strong absorption near 800 cm^−1^ and relatively weak absorption in the 600 cm^−1^ region in IR spectroscopy. Similarly, earlier matrix‐isolation work using alkali and alkaline earth metal atoms with ozone in excess argon gave a strong IR band near 800 cm^−1^ and a weaker IR band near 600 cm^−1^ which were assigned to the antisymmetric O−O stretching (v_3_) and symmetric O<O_2_ bending modes (v_2_), respectively, of the ozonide ion isolated in rare gas matrices in the M^+^O_3_^−^ species.[^17^, ^18^] Furthermore the matrix resonance Raman spectrum for the ozonide ion M^+^O_3_^−^ (M=Li, Na, K, Rb, Cs) exhibited a very strong band from 1004 to 1026 cm^−1^ with its overtone series for the symmetric stretching mode (v_1_).^[15]^ The composition of the absorber in these experiments was determined using scrambled ozone isotope experiments, similar to the discussion for MO_2_ species above. For example, the Ca atom reaction, gave a well resolved sextet for the v_3_ mode which identified a product with a unique O atom and two equivalent O atoms, that is Ca(η^2^−O_3_).^[18]^ Based on these results, the intense v_3_ bands at 868.6 and 828.6 cm^−1^ (frequency ratio=1.0483) and v_2_ bands at 610.5 and 580.3 cm^−1^ (frequency ratio=1.0520) obtained in solid argon in the present study are thus assigned for Hg^16^O_3_ and Hg^18^O_3_, respectively (Figure 1). The isotopically labelled 1 : 1 mixture of ^16^O_2_+^18^O_2_ in Figure 1 (top, spectra (i) to (l)) also shows strong bands at 868.6 and 828.6 cm^−1^ for the terminal O−O subunit in the ozonide group. Appropriate intermediate mixed isotopic species were observed for this *C*_2v_ structured O_3_ subunit.[^15^, ^17^] Additional experiments in solid neon and solid oxygen were performed to support our assignments for the newly produced ozonide species (Tables 2 and S1, Figures S1 and S2).

Having already assigned the new species obtained in our experiments, we focus on the Hg−O species in the following. Previously, a band at 676 cm^−1^ was tentatively assigned to Hg−O by the Snelson group who did not show any IR spectra in their report.^[3]^ Hg was added to argon with 0.5 to 5 % O_3_ all under photolysis from a medium pressure mercury arc lamp in these experiments.^[3]^ Their 676 cm^−1^ band is clearly out of the range based on high‐level calculations of the elusive Hg−O diatomic molecule.[^4^, ^5^] However, its reported 676/642=1.053 ratio for the 16/18 isotopic frequency ratio is appropriate for an Hg−O vibration. The Pt−O molecule exhibits a similar 828.0/784.4=1.056 isotopic frequency ratio in argon matrix.^[22]^ However, as can be seen in Figure 1, there is only a very weak band in our experiments (observed at 678.4 cm^−1^), which increases slightly upon annealing and may have been favored by the higher Hg concentrations in the first work by Snelson group.^[3]^ Our previous work with Hg and (CN)_2_ ^[23]^ shows that electronegative substituents support Hg−Hg bonding, and we tentatively assign this 676 cm^−1^ band instead to the more difficult dimercury oxide species. Mercury‐mercury bonding is considered in a recent review^[24]^ and metal‐metal bonding has been investigated computationally for the entire Zn, Cd, Hg family.^[25]^

### Comparison of HgO with analogous molecules

First, it is remarkable to compare the spectra of the first mercury oxyfluoride FHg−O obtained from the reaction of laser ablated amalgam with OF_2_ ^[13]^ and the simple oxide Hg−O from the present study obtained under the same experimental conditions. A relatively broader band at 637.6 cm^−1^ for the triatomic FHg−O was observed compared to 521.2 cm^−1^ for the diatomic Hg−O in solid argon. The analogous experiments with ^18^O labelling produced a band at 625.2 cm^−1^ for FHg−O and a band at 496.4 cm^−1^ for HgO, with oxygen isotope shifts of 12.4 and 24.8 cm^−1^, respectively. This isotopic shift indicates that both F and O are equally involved in the antisymmetric F−Hg−O stretching, while the Hg−O stretching is almost a pure O motion against the much heavier Hg atom. Finally, calculations at the CCSD(T)/aug‐cc‐pVTZ‐PP level of theory show that the Hg−O bond length is longer in the triatomic (194.9 pm) than in the diatomic (191.9 pm) species.

It is also interesting to compare the frequencies for Pt−O, Au−O and Hg−O as an additional *d* orbital electron is added to this series with the argon matrix frequencies 828.0, 619.2 and 521.2 cm^−1^ which decrease, respectively, where their metal reactivity also decreases in this series.[^22^, ^26^] We can add one more molecule to this series, namely Cs−O at 321.7 cm^−1^,^[17]^ which is surely a highly ionic molecule. This and the small difference between our neon and argon matrix observations for Hg−O suggest a slightly ionic molecule, consistent with the large difference in electronegativities *χ*(Hg)=1.8 and *χ*(O)=3.5.^[27]^

Finally, we compare the Hg−O with the other group 12 metal oxides. In a previous study, laser ablated Zn and Cd atoms have been reacted with O_2_ in argon matrices.^[12]^ The argon matrix gave a resolved zinc isotopic triplet for Zn−O molecules with ^64^Zn−O the most abundant at 48.9 % which produced a sharp 769.2 cm^−1^ band in argon. Its oxygen isotopic ratio 769.2/735.1=1.0464 is slightly less than found for Hg−O (521.2/496.4=1.0500) since the lighter metal atom moves more and the O less in Zn−O than in Hg−O. The 769.2 cm^−1^ band for Zn−O compares to 713.4 cm^−1^ for the MRCI+Q/CBS calculations.^[5]^ Cd gave a sharp absorption at 645.1 cm^−1^ in argon somewhat higher than the high‐level calculation of 598.0 cm^−1^. Note, our Hg−O produced a much smaller number of 521.2 cm^−1^ than the 605 cm^−1^ computed value at the same level of theory. The matrix‐isolated molecules are usually shifted to 10–20 cm^−1^ lower wavenumber,^[11]^ so these comparisons probably reflect less accuracy for the high‐level calculations.^[5]^ Several computational studies comparing group 12 metal oxides and chalcogenides have been documented in the literature.[^4^, ^7^]

## Conclusions

In summary, laser ablated mercury atoms from a dental amalgam target together with O_2_ produced HgO, HgO_2_ and HgO_3_ for the first time, as well as oxygen dissociation products under the matrix isolation conditions at 4 K. Assignments were made using ^16/18^O isotopic substitution experiments supported by CCSD(T) calculations. Our observation of the Hg−O diatomic fundamental frequency at 521.2 cm^−1^ in solid argon, 529.0 cm^−1^ in solid neon and 522.7 cm^−1^ in solid oxygen can be taken as experimental support for the Shepler, Peterson calculated frequency ranges[^4^, ^5^] for the ground state ^1^Σ^+^ Hg−O molecule. Furthermore, the O−O stretching mode of an isosceles triangle Hg‐O_2_ at 1207.1 cm^−1^ as well as the antisymmetric O−O stretching at 868.6 cm^−1^ and the bending at 610.5 cm^−1^ bands of HgO_3_ in solid argon were identified by IR spectroscopy. This work could have applications in atmospheric chemistry, as Hg−O has been proposed as one of the major products of oxidation and degradation of gaseous mercury in the Earth's atmosphere.

## Experimental Section

The matrix‐isolation setup and the laser‐ablation apparatus in our laboratory have been described in detail previously.^[28]^ The preparation of the mercury amalgam/alloy target was also documented.^[13]^ In a typical experiment, laser‐ablated mercury atoms were co‐deposited with 0.02 to 0.3 % oxygen diluted with an excess of neon or argon onto a gold‐plated mirror at 4 K. This low temperature was achieved using a closed‐cycle helium cryostat (Sumitomo Heavy Industries, RDK‐205D). For laser‐ablation, the 1064 nm fundamental of a Nd:YAG laser (Continuum, Minilite II, 10 Hz repetition rate, 50–60 mJ pulse^−1^) was focused onto a rotating amalgam target. The deposition times varied between 60‐ and 180‐min. After deposition, the matrix samples were subjected to annealing to 11 K (neon matrices), 16 K (oxygen matrices) and 25 K (argon matrices) and irradiation by a medium pressure mercury arc streetlamp (λ>220 nm). IR spectra were recorded at a resolution of 0.5 cm^−1^ on a Bruker Vertex 80v spectrometer by using a liquid‐nitrogen‐cooled mercury cadmium telluride (LN‐MCTB) detector. Structural optimizations and frequency calculations were carried out at the Coupled Cluster Single Double and perturbative Triple excitations (CCSD(T))^[29]^ level in conjunction with the augmented triple‐ζ basis sets aug‐cc‐pVTZ for oxygen and the aug‐cc‐pVTZ‐PP^[30]^ valence basis and associated scalar‐relativistic pseudopotential (PP) for mercury using the Molpro 2019 software package.^[31]^

## Conflict of interest

The authors declare no conflict of interest.

1

## Supporting information

As a service to our authors and readers, this journal provides supporting information supplied by the authors. Such materials are peer reviewed and may be re‐organized for online delivery, but are not copy‐edited or typeset. Technical support issues arising from supporting information (other than missing files) should be addressed to the authors.

Supporting Information

## Data Availability

The data that support the findings of this study are available from the corresponding author upon reasonable request.
