# Supplementary material for: Observation and Characterization of the Hg‐O Diatomic Molecule: A Matrix‐Isolation and Quantum‐Chemical Investigation
Source: Chemistry. 2022 Dec 14;29(7):e202202740. doi: 10.1002/chem.202202740 (PMC10107950; doi:10.1002/chem.202202740)
Supplement: Supplementary file 1 — Supporting Information [file CHEM-29-0-s001.pdf]

Chemistry–A European Journal

Supporting Information

Observation and Characterization of the Hg-O Diatomic Molecule: A Matrix-Isolation and Quantum-Chemical Investigation

Lester S. Andrews,* Yetsedaw A. Tsegaw,* Han-Gook Cho, and Sebastian Riedel*

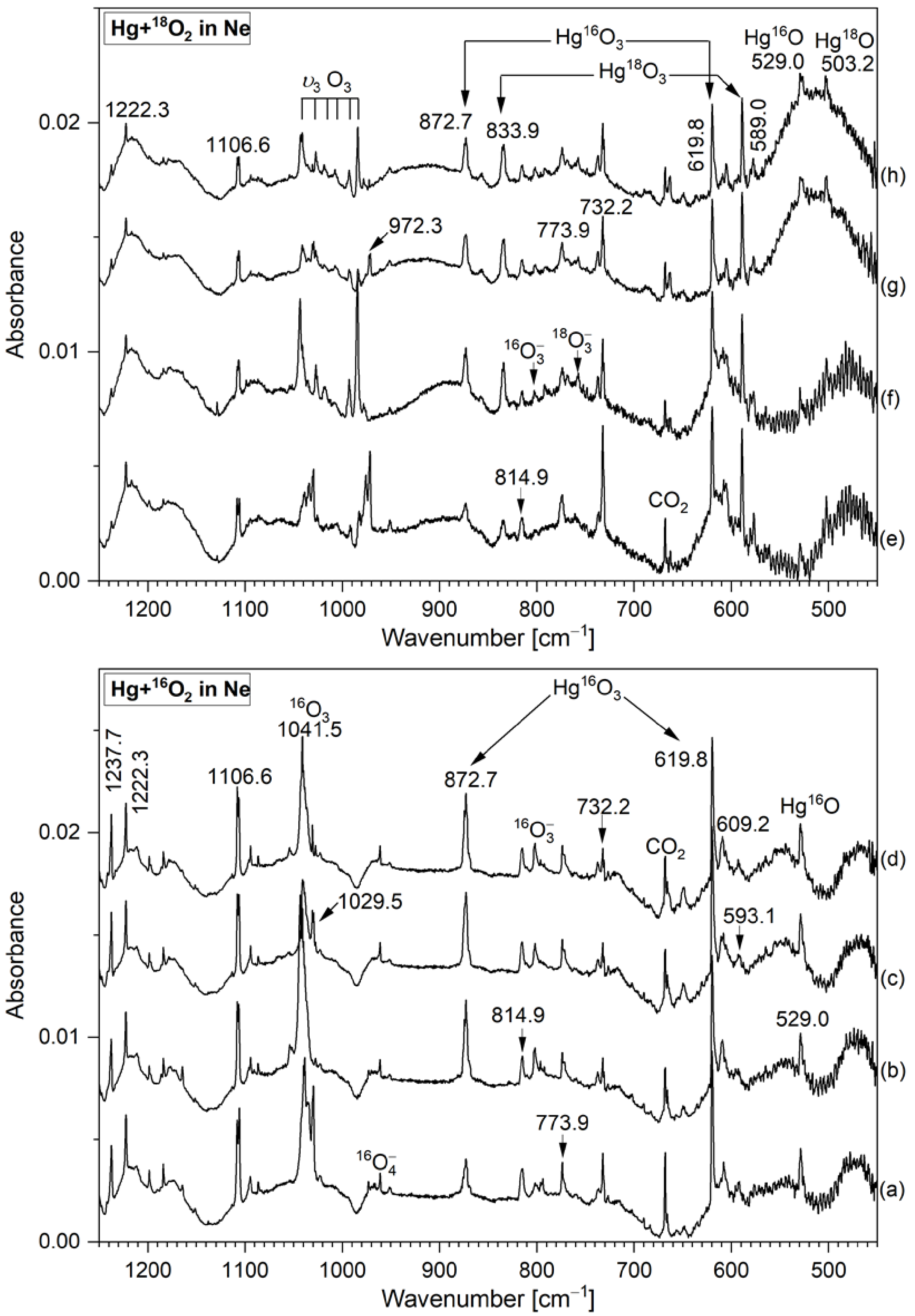

Figure S1: IR spectra of the reaction products from laser ablated Hg co-deposited with 0.02% O_2 (bottom) and 0.02% $^{18}O_2$ (top) in neon at 4 K. Spectra (a, e) after deposition for 60 min at 4 K, (b, f) after annealing to 11 K, (c, g) after 10 min full-arc photolysis with mercury lamp and (d, h) after annealing to 12 K. Note that the top spectra show almost equal isotopic concentrations with $^{18}O_2$ added to $^{16}O_2$ residual in the vacuum system.

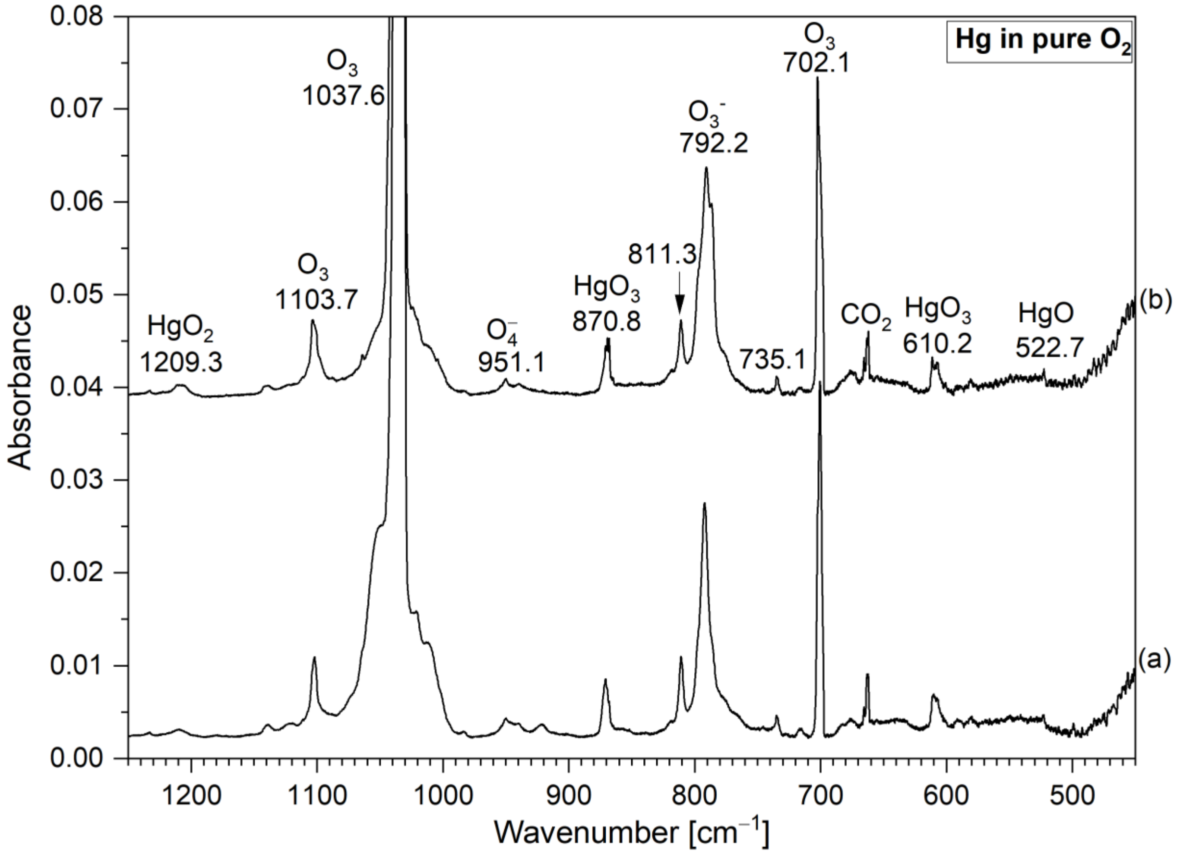

Figure S2: IR spectra of the reaction products from laser ablated Hg deposited in pure oxygen matrix at 4 K. Spectra (a) after deposition for 60 min at 4 K and (b) after annealing to 16 K.

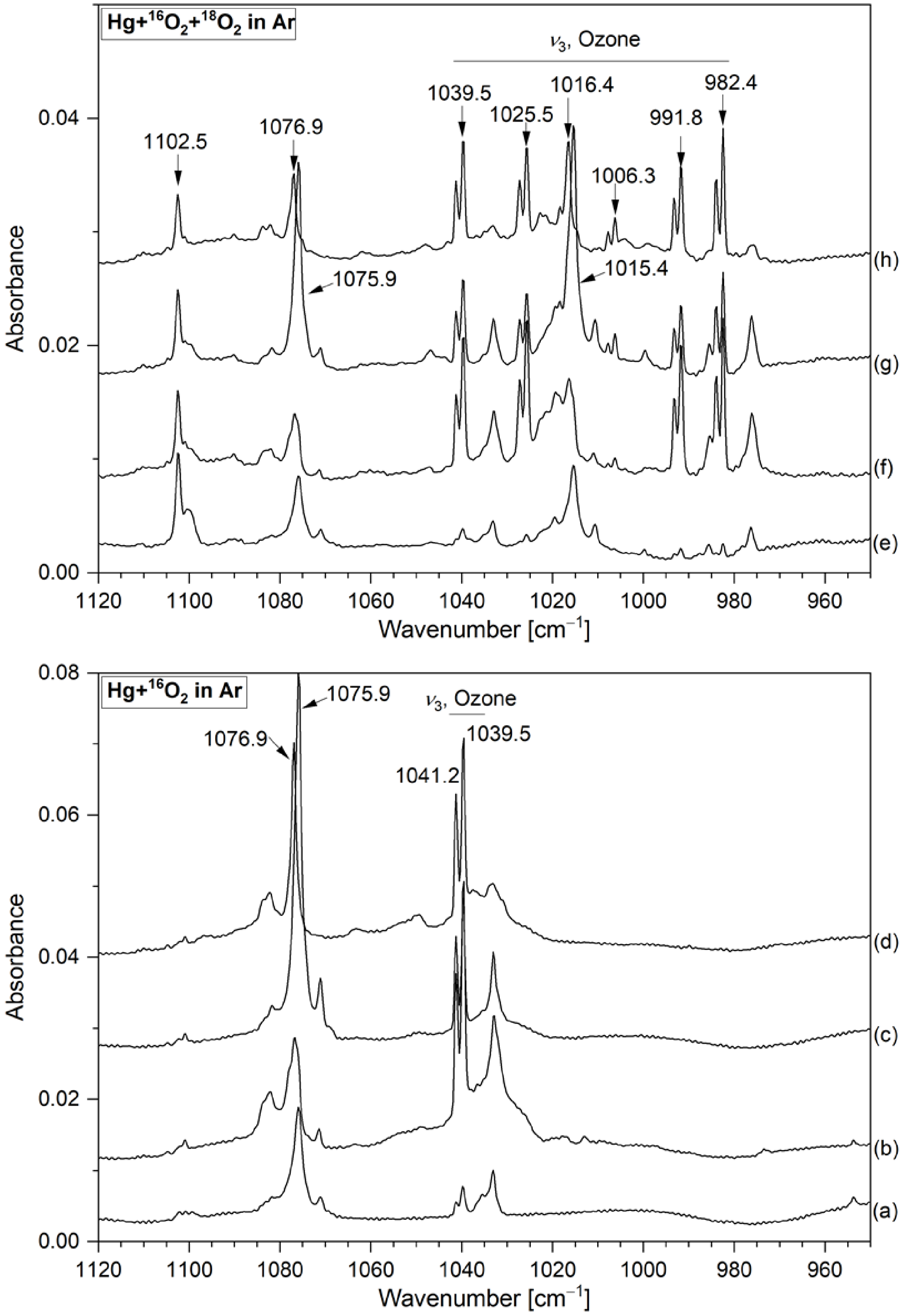

Figure S3: Expanded region of ozone (ν_3): IR spectra of the reaction products from laser ablated Hg co-deposited with 0.3% $^{16}O_2$ (bottom) and 0.3% $^{16}O_2$+$^{18}O_2$ (top) in argon at 4 K. Spectra (a, e) after deposition for 60 min at 4 K, (b, f) after annealing to 25 K, (c, g) after 10 min full-arc photolysis with mercury lamp and (d, h) after annealing to 30 K.

Table S1. Frequencies (cm^{-1}) observed for the reaction products of laser ablated mercury atoms with oxygen trapped in solid argon, neon and oxygen at 4 K.

Argon			Neon			Oxygen	Assignment
$^{16}O_2$	$^{18}O_2$	Ratio (16/18)	$^{16}O_2$	$^{18}O_2$	Ratio (16/18)	$^{16}O_2$	
						1552.4	$(O_2)_n$
1207.1	1139.1	1.0597	1220.0[b]	1159.0[b]	1.0526	1209.3	HgO_2 (ν_1)
1102.7	1102.5[c]		1106.6			1103.7	O_3 (ν_1)
1076.9	1016.4	1.0595					?
1075.9	1015.4	1.0596					?[d]
1041.2	983.9	1.0582	1041.7	983.4	1.0593	1039.1	O_3 (ν_3) site
1039.5	982.4	1.0581	1041.5	982.9	1.0596	1037.6	O_3 (ν_3)
1033.0	976.2	1.0582	1029.5	972.3	1.0588	1034.5	O_3 (ν_3) site
953.8	901.7	1.0578	973.3	919.8	1.0582	951.1	O_4^-
868.6	828.6	1.0483	872.7	833.9	1.0465	870.8	HgO_3 (ν_3)
810.8	770.3	1.0526	814.9	774.5	1.0522	811.3	HgO_3 (ν_3) site
804.3	759.2	1.0594	801.9	757.3	1.0589	797.1	O_3^- site
796.6	752.9	1.0580	796.3	752.3	1.0585	792.2	O_3^-
790.5	746.2	1.0594	792.5	748.3	1.0591	786.7	O_3^- site
767.6			773.9				?
737.0	736.8	1.0003	732.2	732.2	1.0000	735.1	?
703.5						702.1	O_3 (ν_2)
678.4						675.5	Hg-HgO?
610.5	580.3	1.0520	619.8	589.0	1.0523	610.2	HgO_3 (ν_2)
599.4	569.5	1.0525	609.2	577.4	1.0551		HgO_3 (ν_2) site[e]
590.0	561.3	1.0511	593.1	564.6	1.0505	591.6	HgO_3 (ν_2) site
521.2	496.4	1.0500	529.0	503.2	1.0513	522.7	HgO
499.3	474.9	1.0514	497.8	477.3	1.0429	499.3	-O-Hg-O-Hg-[f]

[a] References: for HgO, HgO_2 and HgO_3 see the main text; for O_3 (see Table S_2); for O_2, O_3^- and O_4^- (M. Zhou, J. Hacaloglu, L. Andrews, *J. Chem. Phys*. **1999**, 110, 9450– 9456; M. E. Jacox, D. E. Milligan, *J. Mol. Spectrosc*. **1972**, 43, 148–167; M. E. Jacox, D. E. Milligan, *Chem. Phys. Lett*, **1972**, 14, 518–521; G. V. Chertihin and L. Andrews, *J. Chem. Phys*. **1998**, 108, 6404 – 6407). [b] Broad bands. [c] Not reproducible. [d] See main text. [e] Tentative assignment. [f] Broad bands assigned to polymeric Hg-O.

Table S2. ν_3 absorptions of ozone (cm^{-1}) obtained in argon and neon matrices.

	Argon				Neon		
	This work	Ref. [1]	Ref. [2]	Ref. [3]	This work	Ref. [4]	Ref. [5]
$^{6}O^{16}O^{16}O$	1041.2<br>1039.5	1041.25<br>1039.58	1039.69	1040.0	1041.7<br>1041.5	1039.7<br>1038.5	1039.9<br>1038.7
$^{18}O^{16}O^{16}O$	1027.2<br>1025.5	1027.28<br>1025.61	1025.63	1026.2	1026.3<br>1024.8	1025.9<br>1024.7	1026.1<br>1024.9
$^{18}O^{16}O^{18}O$	1018.3<br>1016.4	1018.38<br>1016.69	1016.70	1017.1	1017.4<br>1016.1	1017.0<br>1015.8	1017.2<br>1016.0
$^{16}O^{18}O^{16}O$	1007.7<br>1006.3	1007.76<br>1006.18	1006.17	1006.5	1006.5<br>1005.1	1006.2<br>1005.0	1006.4<br>1005.2
$^{18}O^{18}O^{16}O$	993.3<br>991.8	993.20<br>991.62	991.63	992.0	992.1<br>990.9	991.7<br>990.6	992.0<br>990.8
$^{18}O^{18}O^{18}O$	983.9<br>982.4	983.97<br>982.37	982.35	982.8	983.4<br>982.9	982.6<br>981.4	982.7<br>981.5

[1]. P. Brosset, R. Dahoo, B. Gauthier-Roy, L. Abouaf-Marguin, A. Lakhlifi, *Chem. Phys*. **1993**, 172, 315.

[2]. D.W. Green and K.M. Ervin, *J. Mol. Spectry*. 88, **1981**, 51.

[3]. L. Andrews and R. C. Jr. Spiker, *J. Phys. Chem*., **1972**, 76, 3208.

[4]. M. Zhou, J. Hacaloglu, L. Andrews, *J. Chem. Phys*. **1999**, 110, 9450– 9456

[5]. W. E. Thompson, M. E. Jacox, *J Chem Phys*. **1989**, 91, 3826–3837
